# DDX5 promotes gastric cancer cell proliferation *in vitro* and *in vivo* through mTOR signaling pathway

**DOI:** 10.1038/srep42876

**Published:** 2017-02-20

**Authors:** Cheng Du, Dan-qi Li, Na Li, Li Chen, Shi-sen Li, Yang Yang, Ming-xiao Hou, Man-jiang Xie, Zhen-dong Zheng

**Affiliations:** 1 Department of Oncology, General Hospital of Shenyang Military Area Command, Shenyang 110840, P. R. China; 2 Institute of Functional Molecules, Shenyang University of Chemical Technology, Shenyang 110142, P. R. China; 3 Department of Gynaecology and Obstetrics, First Affiliated Hospital, Jilin University, Jilin 130021, P. R. China; 4 Department of Aerospace Medicine, The Fourth Military Medical University, Xi’an 710032, P. R. China; 5 Department of Digestive Surgery, Xijing Hospital of Digestive Diseases, The Fourth Military Medical University, Xi’an 710032, China; 6 Department of Oncology, Xijing Hospital, The Fourth Military Medical University, Xi’an 710032, P. R. China; 7 Rescue Center of Severe Wound and Trauma of PLA, General Hospital of Shenyang Military Area Command, Shenyang 110840, P. R. China

## Abstract

DEAD (Asp-Glu-Ala-Asp) box helicase 5 (DDX5) is an ATP-dependent RNA helicase that is overexpressed in various malignancies. Increasing evidence suggests that DDX5 participates in carcinogenesis and cancer progression via promoting cell proliferation and metastasis. However, the functional role of DDX5 in gastric cancer is largely unknown. In this study, we observed that DDX5 was significantly up-regulated in gastric cancer tissues compared with the paired adjacent normal tissues. The expression of DDX5 correlated strongly with Ki67 index and pathological stage of gastric cancer. *In vitro* and *in vivo* studies suggested that knockdown of DDX5 inhibited gastric cancer cell proliferation, colony formation and xenografts growth, whereas ectopic expression of DDX5 promoted these cellular functions. Mechanically, DDX5 induced gastric cancer cell growth by activating mTOR/S6K1. Treatment of everolimus, the specific mTOR inhibitor, significantly attenuated DDX5-mediated cell proliferation. Interestingly, the expression of DDX5 and p-mTOR in gastric cancer tissues demonstrated a positive correlation. Taken together, these results revealed a novel role of DDX5 in gastric cancer cell proliferation via the mTOR pathway. Therefore, DDX5 may serve as a therapeutic target in gastric cancer.

Gastric cancer is one of the most common cancers worldwide. New cases of gastric cancer numbered 951,600 in 2012, with deaths estimated at 723,100^1^. With the development of precision medicine in oncology, much progress has been made in the diagnosis and treatment of gastric cancer^2^. However, the overall survival rate of gastric cancer remains to be improved, partly due to limited therapeutic targets^3^. Therefore, it is of great importance to explore the molecular mechanisms of gastric cancer progression and identify new therapeutic targets.

DEAD (Asp-Glu-Ala-Asp) box helicase 5 (DDX5) is an ATP-dependent RNA helicase that is overexpressed in various malignancies, such as prostate cancer, breast cancer, colon cancer, non-small cell lung cancer, and glioma^4^,^5^,^6^,^7^,^8^. DDX5 act as transcriptional co-regulators with multiple transcription factors and participates in the development and progression of cancer^9^,^10^. Specifically, the expression of DDX5 strongly increases during the stepwise transition from polyp to adenoma and adenocarcinoma in colon^7^. Moreover, DDX5 form complexes with β-catenin promoting the transcription of several proto-oncogenes, including c-Myc, cyclin D1, c-jun, and fra-1^5^,^7^. In breast cancer, DDX5 is frequently amplified. It is required for cell proliferation by controlling the transcription of genes expressing DNA replication proteins in cells harboring DDX5 amplification^11^. In glioma, DDX5 binds with NF-kB p50 and enhances its nucleus accumulation and transcriptional activity, leading to increased tumor growth^8^.

Accumulating evidence suggests that DDX5 is involved in carcinogenesis and progression, nevertheless, the functional role of DDX5 in gastric cancer is still unknown. In the present study, we determined the elevated expression of DDX5 in gastric cancer tissues compared with the matched normal tissues. We further identified the role of DDX5 in promoting gastric cancer cell growth *in vitro* and *in vivo* through lentivirus-mediated DDX5 up- or down-regulation models. Finally, we found that DDX5 induced gastric cancer cell growth via mTOR signaling pathway. Taken together, these findings suggest that DDX5 plays a pivotal role in gastric cancer cell proliferation and might serve as a potential therapeutic target.

## Methods

### Cell Culture

Human gastric cancer cells (NCI-N87 and KATO III) were purchased from the Shanghai Institute of Cell Biology, Chinese Academy of Sciences (Shanghai, China). These cells were cultured in DMEM or RPMI 1640 medium with 10% fetal bovine serum.

### Tissue specimens

This study was approved by the Research Ethics Committee of General Hospital of Shenyang Military Area Command. All methods for humans were performed in accordance with the relevant guidelines and regulations. A total of 65 fresh primary cancer and paired adjacent normal tissue specimens were collected from gastric cancer patients in Xijing Hospital of Digestive Diseases and General Hospital of Shenyang Military Area Command. Tumor staging was determined according to the American Joint Committee on Cancer criteria. Informed consent was obtained from each patient before study. None of these patients underwent preoperative chemotherapy and/or radiation therapy.

### Real-time PCR

Total RNA was extracted from gastric cancer tissues and the matched adjacent normal tissues using Trizol reagent (Invitrogen, Carlsbad, CA, USA). 1 μg of total RNA from each sample were reverse transcribed into cDNA using PrimeScript™ RT Master Mix Kit (Takara, Dalian, China). Real-time PCR was performed using SYBR Premix Ex Taq II Kit (Takara, Dalian, China) according to the instructions of the manufacturer. The specific primers used were as follows: DDX5 sense, 5′-GCCGGGACCGAGGGTTTGGT-3′ and antisense 5′CTTGTGCTGT GCGCCTAGCCA-3′; GAPDH sense, 5′-GGAAGGTGAAGGTCGGAG TCA-3′ and antisense 5′-GTCATGATGGCAACAATATCCACT-3′.

### Immunohistochemistry

Immunohistochemistry (IHC) analysis was performed as described previously^12^. The paraffin-embedded sections were deparaffinized in xylene, rehydrated in descending percentages of ethanol and heated in citrate buffer (pH 6.0) for antigen retrieval. After washing steps, slides were blocked with 3.0% hydrogen peroxide and 10% goat serum and incubated with a primary antibody at 4 °C overnight. The following antibodies were used: rabbit anti-DDX5 antibody (1:500; Abcam, MA, USA), rabbit anti-Ki67 (1:400; Cell signaling technology, MA, USA). After the sequential incubation with biotinylated secondary antibody, streptavidin-horseradish peroxidase complex and diaminobenzidine (DAB), the slides were counterstained with hematoxylin, dehydrated, and mounted. Finally, sections were observed and imaged under light microscope. DDX5 scores (0–300) were calculated as the staining intensity (0, 1, 2, or 3) × the staining extent (0–100%).

### Lentivirus infection

Stable overexpression of DDX5 was carried out using the lentiviral expression system (Genecopoeia, Rockville, MD, USA) according to the manufacturer’s instructions. The ORF of DDX5 was cloned into pReciever-LV105 (Genecopoeia). Cells transfected with vector were used as negative control. Knockdown of DDX5 was achieved using pGV-112-DDX5-shRNA lentiviral expression plasmid (Genechem, Shanghai, China). Cells transfected with Control-shRNA were used as control. Following transfection, NCI-N87 and KATO III cells were selected using 0.5 ug ⁄mL puromycin (Sigma, CA, USA).

### Western blot analysis

Western blot was performed as previously reported^13^. Briefly, whole-cell lysates were prepared and protein concentration was estimated using a BCA Protein Assay kit (Pierce, Rockford, MA, USA). The immune-blots were probed with primary antibodies overnight at 4 °C followed by secondary antibodies for 1 hr. The following primary antibodies were used according to the manufacturer’s protocols: rabbit anti-DDX5, anti-mTOR, anti-p-mTOR (phospho S2448), anti-S6K1, anti-p-S6K1 (phospho T389), mouse anti-β-actin, peroxidase conjugated goat anti-rabbit or anti-mouse IgG (all from Abcam). The blots were visualized using enhanced chemiluminescence detection kit (Thermo). The bands were scanned and quantified by densitometric analysis using Image J software (National Institutes of Health, Bethesda, MD, USA).

### CCK-8 assay

Cell proliferation was measured by CCK-8 assay. Brifely, lentivirus infected cells were plated in 96-well plates at a density of 3,000 cells per well. At various time points (24, 48, 72 and 96 h), 10 μl of CCK-8 solution was added to each well and incubated at 37 °C for 2 h, and then the absorbance was measured at 450 nm. The experiment was performed independently in triplicate.

### Colony formation assay

Two hundred gastric cancer cells were seeded into 6 cm plates and incubated for 12 days. Cells were then fixed with 4% paraformaldehyde and dyed with crystal violet. Colonies were counted and photographed. The experiment was performed independently in triplicate.

### Animal studies

Animal studies were conducted in accordance with the Animal Research: Reporting *In Vivo* Experiments (ARRIVE) guidelines with the approval of the Animal Care and Use Committee of Fourth Military Medical University. NCI-N87 cells were infected with DDX5 or DDX5-shRNA and their negative control vector. Stable infected cells (3 × 10^6^) were subcutaneously inoculated in the lower rear flank of 5-week-old BALB/c nude mice. On day 30 after implantation, tumors were harvested and weighed. The tumor volume was calculated (volume = length × width^2^ × 0.52). IHC analysis was performed as previously described to determine the expression of DDX5 and Ki67.

### Statistical analysis

All statistical analyses were carried out using the SPSS 18.0 statistical software package. Numerical data were presented as mean ± standard error. Two-tailed Student’s t-test was performed. The expression of DDX5 in gastric cancer tissues of different pathological stages were compared by Mann-Whitney U test. The association between DDX5 and Ki67 and p-mTOR was evaluated by correlation analysis. P value less than 0.05 in all cases was considered statistically significant.

## Results

### Overexpression of DDX5 in gastric cancer tissues

**Figure 1:**
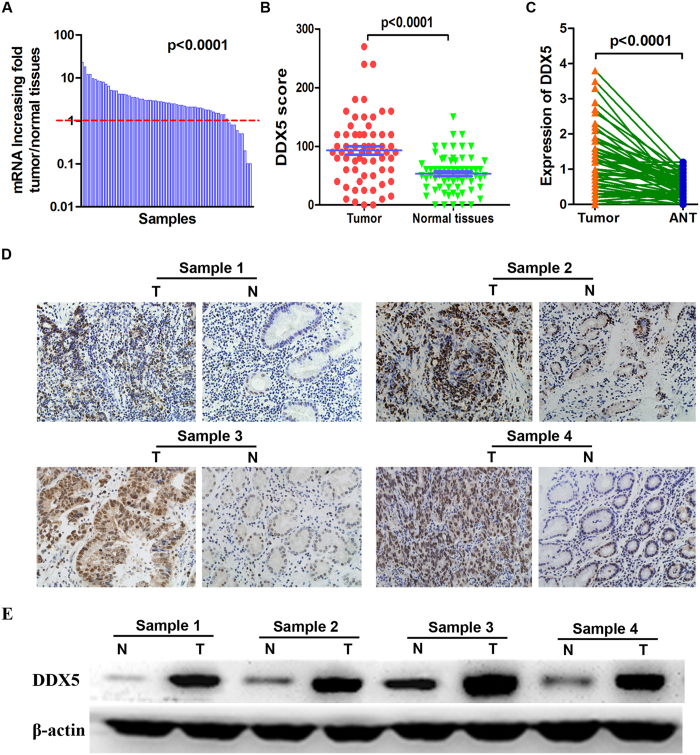
Overexpression of DDX5 in gastric cancer and adjacent normal tissues. (**A**) Relative expression of DDX5 mRNA in 65 gastric cancer and paired adjacent normal tissues as determined by qRT-PCR. Increased expression of DDX5 mRNA (≥2 fold) as compared with normal tissue was observed in 46 (70.7%) of the 65 paired samples. (**B**) Expression of DDX5 protein determined by IHC analysis. The IHC score of DDX5 was calculated as the staining intensity (0, 1, 2, or 3) × the staining extent (0–100%). (**C**) Expression of DDX5 protein determined by Western blot analysis. (**D**) Representative images of IHC staining of DDX5 in gastric cancer (**T**) and adjacent normal tissues (**N**). (**E**) Representative images of immune-blots of DDX5 in gastric cancer and adjacent normal tissues (ANT).

**Figure 2:**
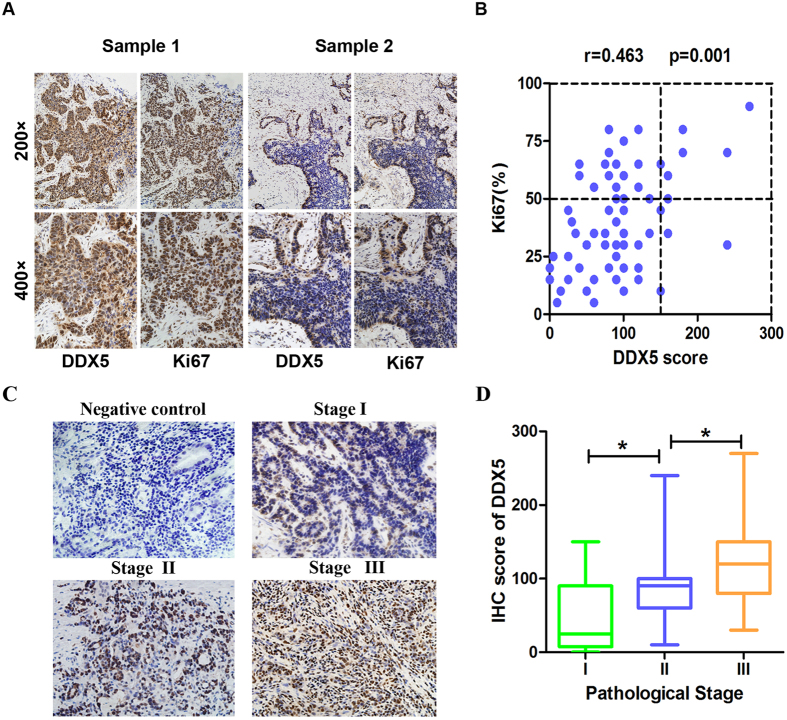
Immunohistochemical analysis of DDX5 and Ki67 in gastric cancer tissues. (**A**) Representative images of IHC staining of DDX5 and Ki67 in 65 gastric cancer tissues. (**B**) Correlation analysis of the expression of DDX5 and Ki67 in gastric cancer tissues. Each point represents one gastric cancer specimen. (**C**) IHC staining of DDX5 in gastric tissues of different pathological stages. For negative control, sections were immune-stained with the anti-IgG antibody instead of anti-DDX5. (**D**) Expression of DDX5 increases as gastric cancer progresses to more advanced stages. *p < 0.05, Mann-whitney U test.

First, we examined the expression of DDX5 in gastric cancer tissues. Real-time PCR analysis demonstrated increased expression of DDX5 mRNA (≥2 fold) as compared to normal tissue in 46 (70.7%) of the 65 gastric cancer samples (Fig. 1A). This was further supported by the results of IHC and western blot analysis, which showed more expression of DDX5 protein in gastric cancer tissues (Fig. 1B–E). We next investigated the association between DDX5 and Ki67 index. As shown in Fig. 2A,B, the expression of DDX5 significantly correlated with that of Ki67 in gastric cancer tissues, indicating a potential role of DDX5 in gastric cancer proliferation. Moreover, we found that the expression of DDX5 increased significantly as gastric cancer progressed to more advanced stage (Fig. 2C,D).

### DDX5 promotes gastric cancer cell growth *in vitro*

**Figure 3:**
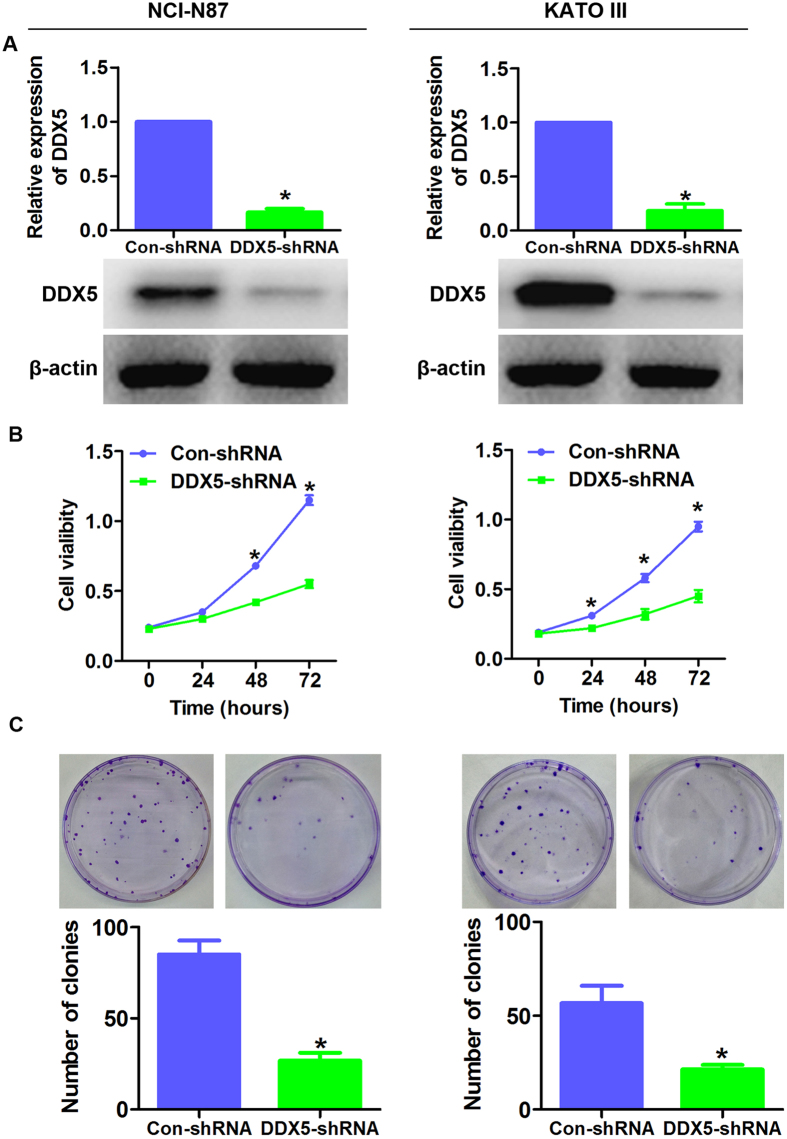
Silencing of DDX5 inhibits gastric cancer cell proliferation. (**A**) Western blot analysis of DDX5 in NCI-N87 and KATO III cells transfected with DDX5-shRNA or Control-shRNA. (**B**) CCK-8 analysis of gastric cancer cells infected with the indicated lentivirus. 3 × 10^3^ cells were seeded in 96 well plates and cultured for the indicated hours (**C**). Colony formation analysis of gastric cancer cells. 200 cells were seeded in 6 cm plates and cultured for 12 days. *p < 0.05.

**Figure 4:**
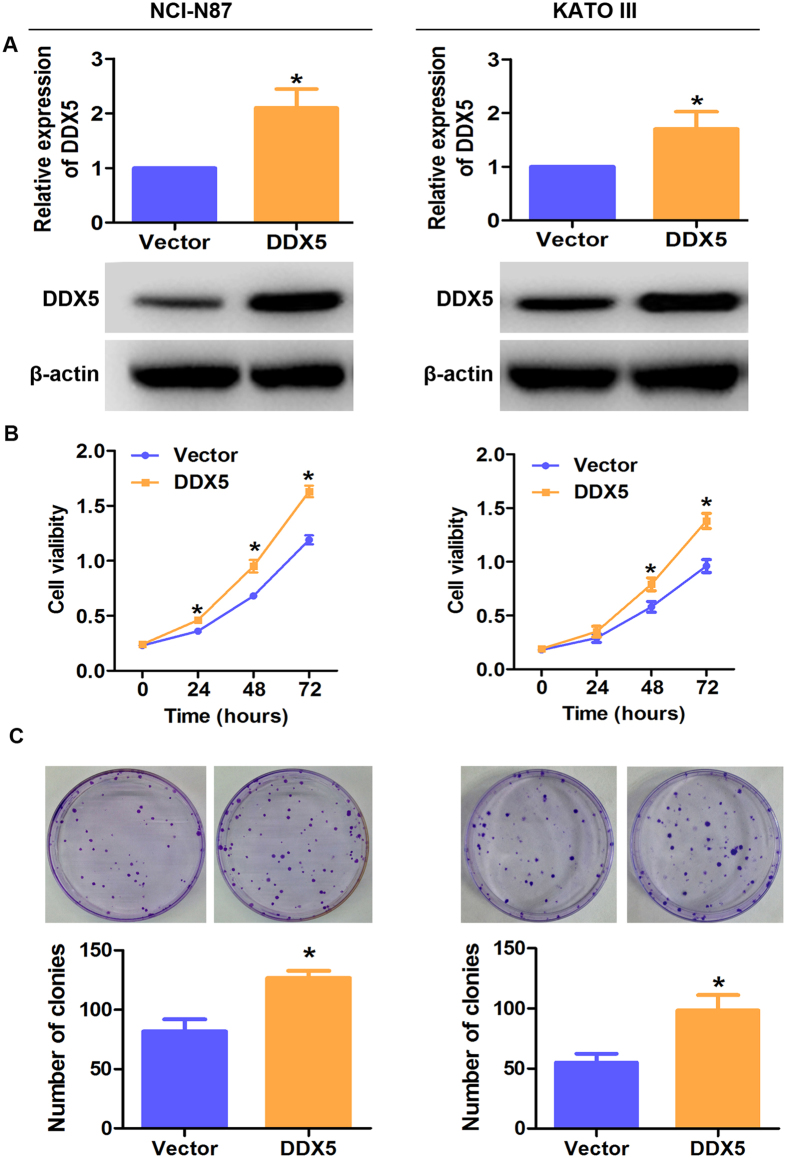
Overexpression of DDX5 promotes gastric cancer cell proliferation. (**A**) Western blot analysis of DDX5 in NCI-N87 and KATO III cells transfected with DDX5 or vector. (**B**) CCK-8 analysis of gastric cancer cells infected with the indicated lentivirus. 3 × 10^3^ cells were seeded in 96 well plates and cultured for the indicated hours (**C**). Colony formation analysis of gastric cancer cells. 200 cells were seeded in 6 cm plates and cultured for 12 days. *p < 0.05, Mann-whitney U test.

To assess the functional role of DDX5 in gastric cancer cells, we established lentivirus mediated DDX5 up- and down-regulation systems in NCI-N87 and KATO III cell lines. As shown in Fig. 3A, DDX5-shRNA efficiently silenced the expression of DDX5 in both cell lines. Consequently, cell growth was significantly inhibited in DDX5-shRNA transfected cells than that in Control-shRNA transfected cells as determined by CCK-8 and colony formation assays (Fig. 3B,C). On the contrary, up-regulation of DDX5 dramatically increased cell proliferation and colony formation (Fig. 4A–C). Collectively, these data suggested that DDX5 played an important role in gastric cancer cell growth *in vitro*.

### DDX5 promotes gastric cancer cell growth *in vivo*

**Figure 5:**
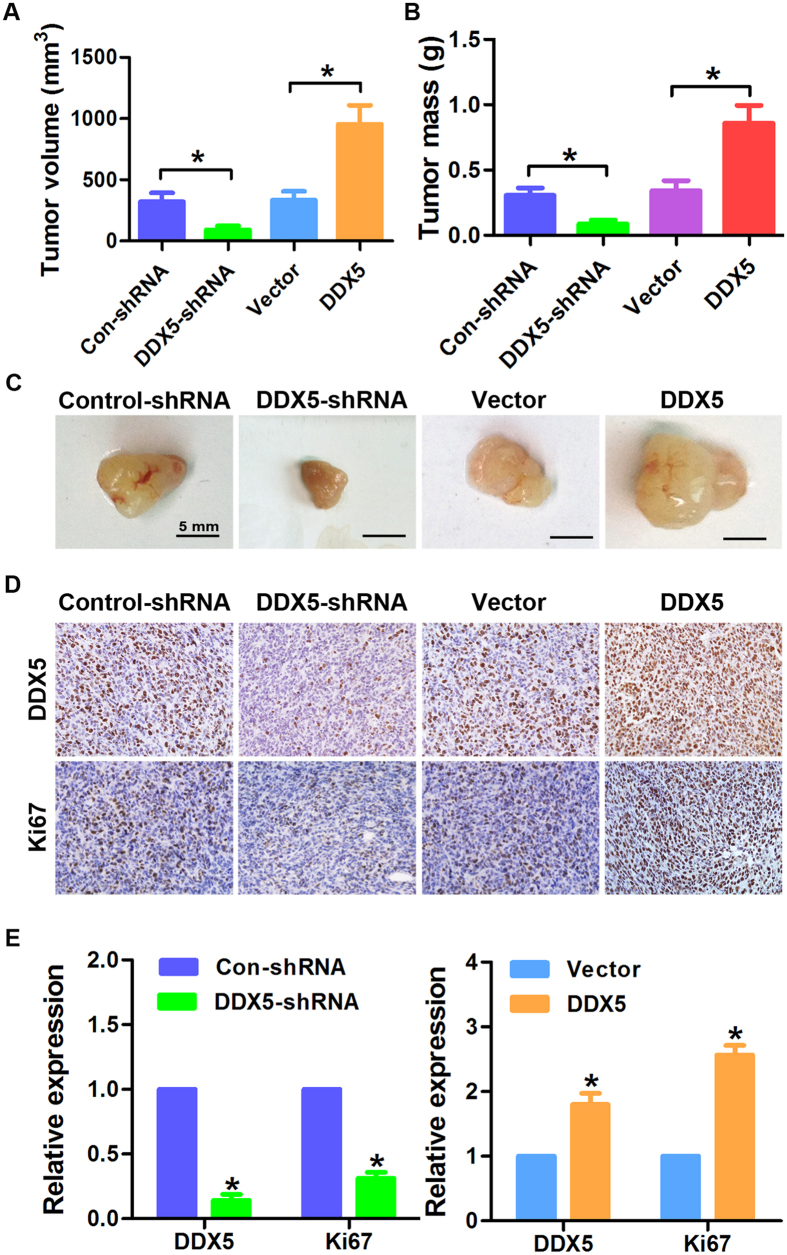
The effects of DDX5 modulation on the growth of xenografts in nude mice. NCI-N87 cells infected with DDX5 or Vector (100 μl; 3 × 10^6^ cells) were implanted subcutaneously into Balb/c-nude mice to form xenografts. After 30 days, the xenografts were harvested and processed to immune-staining. (**A,B**) Mean tumor volume and tumor mass of xenografts in different groups. (**C**) Representative images of xenografts. (**D,E**) Immunohistochemistry analysis of DDX5 and Ki67 in xenografts of the indicated groups. *p < 0.05.

To further investigate the influence of DDX5 up- or down-regulation on gastric cancer growth *in vivo*, we established the subcutaneous xenograft in nude mice using NCI-N87 cells. As shown in Fig. 5A–C, the tumor volume and weight in DDX5-shRNA group were significantly smaller than that in Control-shRNA group. Conversely, up-regulation of DDX5 significantly increased growth of the xenografts. The IHC staining of Ki67 further revealed that knockdown of DDX5 inhibited gastric cancer proliferation *in vivo*, while ectopic expression of DDX5 exhibited the opposite effects (Fig. 5D,E).

### DDX5 stimulates gastric cancer cell proliferation via mTOR signaling pathway

**Figure 6:**
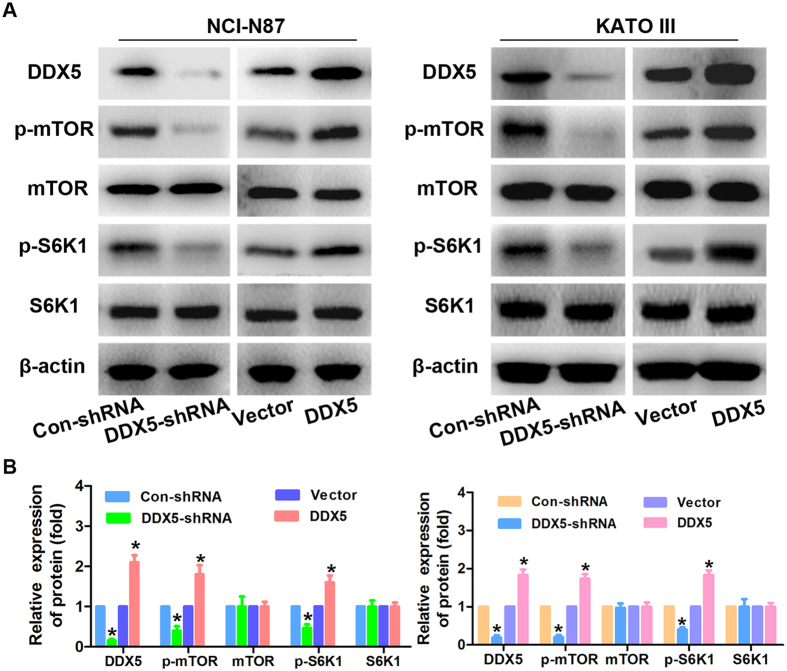
DDX5 regulates the mTOR signaling in gastric cancer cells. (**A**) Western blot analysis of the expression of DDX5, p-mTOR(S2448), mTOR, p-S6K1(T389) and S6K1 in the indicated gastric cancer cells in response to DDX5 up-regulation or down-regulation. β-actin was used as loading control. (**B**) Relative quantification of the indicated proteins by densitometric analysis of the bands. All the experiments were performed in triplicate. *p < 0.05.

**Figure 7:**
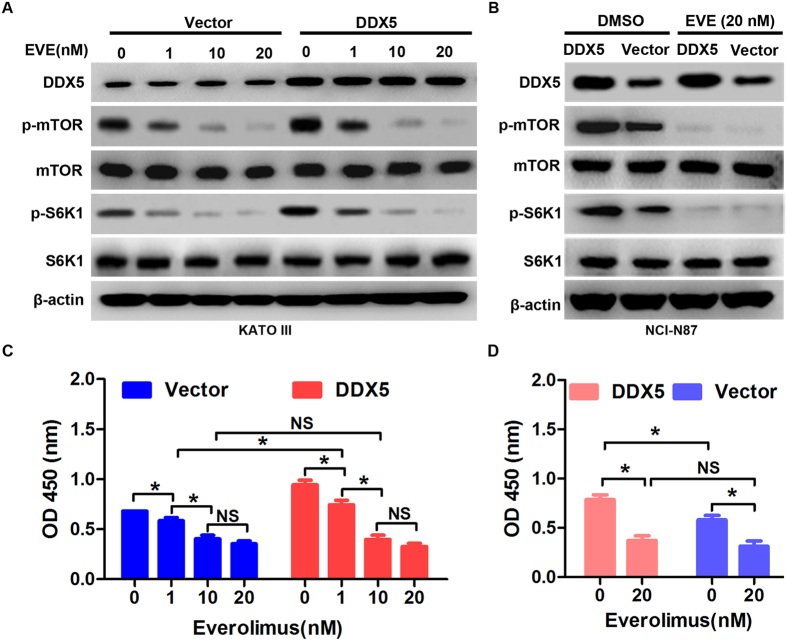
Suppression of mTOR signaling abrogates DDX5-induced cell proliferation. (**A,B**) Western blot analysis of DDX5, p-mTOR(S2448), mTOR, p-S6K1(T389) and S6K1 in DDX5 or Vector transfected KATO III and NCI-N87 cells incubated with the indicated concentration of everolimus (EVE) or DMSO for 48 hr. β-actin was used as loading control. (**C,D**) CCK-8 analysis of gastric cancer cell proliferation in the presence or absence of everolimus for 48 hr (*p < 0.05; NS, no significance).

**Figure 8:**
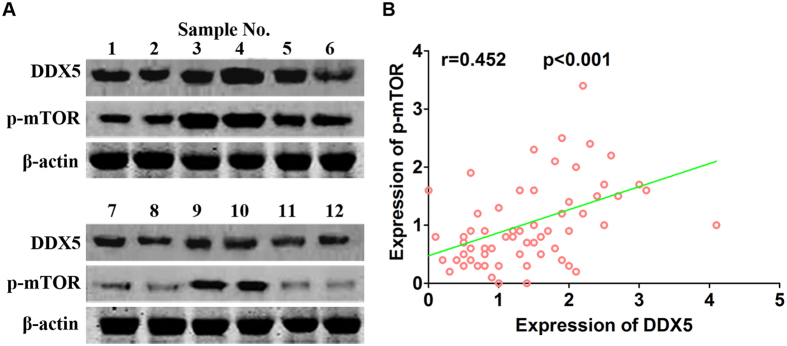
Expression of DDX5 positively correlated with p-mTOR in gastric cancer specimens. (**A**) Representative images of immunoblots of DDX5 and p-mTOR(S2448) in 65 gastric cancer specimens. β-actin was used as loading control. (**B**) Correlation analysis of the relative expression of DDX5 and p-mTOR(S2448).

As mTOR signaling pathway plays an important role in cancer proliferation^14^, we investigated whether DDX5-induced gastric cancer cell proliferation was mediated through mTOR pathway. Western blot analysis revealed that down-regulation of DDX5 significantly inhibited the phosphorylation of mTOR and its downstream molecule S6K1, while up-regulation of DDX5 increased the activity of mTOR/S6K1 signaling (Fig. 6A,B). However, modulation of DDX5 did not alter the total protein levels of mTOR and S6K1 (Fig. 6A,B). Next, we studied the effects of overexpression of DDX5 on mTOR/S6K1 signaling in the presence of everolimus, a specific mTOR inhibitor. As shwon in Fig. 7A, everolimus significantly abrogated DDX5-induced phosphorylation of mTOR and S6K1 in a dose dependent manner. The activity of mTOR/S6K1 signaling was almost totally inhibited by 20 nM everolimus in both cell lines, regardless of the expression level of DDX5 (Fig. 7A,B and Fig. S1). In line with this, DDX5-induced cell proliferation was also abolished by 20 nM everolimus as determined by CCK-8 assay (Fig. 7C,D). Finally, we analyzed the expression of DDX5 and p-mTOR in clinical specimens. As revealed by western blot analysis, the expression of DDX5 and p-mTOR demonstrated a strong correlation in gastric cancer tissues. (Fig. 8A,B). These results strongly suggested that DDX5 induced gastric cancer cell proliferation is mainly mediated via mTOR/S6K1 signaling pathway.

## Discussion

DDX5 is first identified as a RNA helicase, participating in nearly all aspects of RNA metabolism, such as miRNA maturation, ribosome biogenesis and mRNA splicing^15^. Recent studies suggest that DDX5 is frequently overexpressed in a variety of malignancies and contributes to cancer development and progression^16^,^17^,^18^. In NSCLC and glioma, DDX5 was significantly overexpressed in cancerous tissues compared with normal adjacent tissues and predicted poor prognosis^5^. In breast cancer, DDX5 correlated strongly with Ki67, a nuclear marker for cancer cell proliferation indicating poor prognosis and high invasiveness^19^. In consistence with these studies, we also observed elevated expression of DDX5 in gastric cancer relative to the matched normal tissues. Moreover, high expression of DDX5 was strongly associated with high Ki67 index and advanced pathological stage. Inspired by these findings, we further explored the role of DDX5 in gastric cancer proliferation *in vitro* and *in vivo*. As expected, knockdown of DDX5 inhibited gastric cancer cells proliferation *in vitro* and tumorigenesis *in vivo*. Conversely, ectopic expression of DDX5 showed the opposite effects. These findings were consistent with the previous reports that DDX5 was a key regulator in promoting cell proliferation in various tumors^5^,^8^,^11^,^20^,^21^.

Many reports identified DDX5 as transcriptional co-regulator to promote cancer cell proliferation. In colon cancer and NSCLC, DDX5 interacts with β-catenin and stimulates the transcription of downstream molecules, including c-Myc and cyclinD1^5^,^7^,^22^. DDX5 also function as co-activator of estrogen receptor, androgen receptor, E2F1 and NFκB, facilitating cell proliferation in breast cancer, prostate cancer and glioma^4^,^6^,^8^,^11^,^23^. These studies indicate that the molecular mechanism of DDX5 induced proliferation is cellular context dependent.

The mammalian target of rapamycin (mTOR) is a key downstream effecter of several signaling pathways that is involved in cancer progression, including PI3K/Akt and AMPK pathway^14^,^24^. As a protein kinase, mTOR could phosphorylate key components of the protein synthesis machinery, such as S6 kinase (S6K1)^25^. It has been determined that the activated mTOR pathway is critical for cell proliferation and predicts poor prognosis in gastric cancer^26^,^27^,^28^,^29^. Therefore, we investigated the possible role of mTOR signaling in DDX5 mediated gastric cancer cell growth. We found that up-regulation of DDX5 activated mTOR/S6K1 and led to increased cell proliferation. On the contrary, down-regulation of DDX5 attenuated this pathway and resulted in suppression of cell growth. To further prove these findings, we studied the effects of everolimus on Vector or DDX5 overexpressed gastric cancer cells. The data suggested that everolimus displayed dose dependent effect on mTOR/S6K1 activity and cell proliferation. Moreover, treatment of 20 nM everolimus dramatically abolished DDX5-induced mTOR/S6K1 activation and gastric cancer cell proliferation. Intriguingly, we also observed a positive correlation between DDX5 and p-mTOR in gastric cancer tissues. These data indicates that mTOR/S6K1 serves as downstream effecter of DDX5 in promoting gastric cancer growth. Our findings were in agreement with the previous reports that mTOR pathway was an essential mediator of the DDX5-dependent cell growth in prostate cancer^30^. This has implications for gastric cancer treatment. Currently, there is no effective target for gastric cancer except for HER2, which is over-expressed in only about 12% to 27% of gastric cancers^31^. We observed frequently high expression of DDX5 and its strong association with p-mTOR in gastric cancer. Therefore, targeting DDX5/mTOR/S6K1 might be a novel approach for the treatment of gastric cancer. In fact, DDX5 is druggable and serves as direct targets of (-)-Epigallocatechin-3-gallate in gastric cancer and resveratrol in prostate cancer^30^,^32^. Treatment with these drugs resulted in significant degradation of DDX5 and suppression of the cell growth.

In conclusion, our study demonstrates that DDX5 promote gastric cancer cell proliferation via mTOR signaling. Future work is clearly warranted to elucidate mechanistically how DDX5 regulates this pathway, and to validate the utility of DDX5 for gastric cancer therapy.

## Additional Information

**How to cite this article**: Du, C. *et al*. DDX5 promotes gastric cancer cell proliferation *in vitro* and *in vivo* through mTOR signaling pathway. *Sci. Rep.* **7**, 42876; doi: 10.1038/srep42876 (2017).

**Publisher's note:** Springer Nature remains neutral with regard to jurisdictional claims in published maps and institutional affiliations.

## Supplementary Material

Supplementary Information
