# Supplementary material for: DDX5 promotes gastric cancer cell proliferation in vitro and in vivo through mTOR signaling pathway
Source: Sci Rep. 2017 Feb 20;7:42876. doi: 10.1038/srep42876 (PMC5316961; doi:10.1038/srep42876)
Supplement: Supplementary Information [file srep42876-s1.pdf]

DDX5 promotes gastric cancer cell proliferation in vitro and in vivo through mTOR signaling pathway

Cheng Du[1*], Dan-qi Li[2*], Na Li[3]*, Li Chen[4]*, Shi-sen Li[5], Yang Yang[6], Ming-xiao Hou[7], Man-jiang Xie[4] and Zhen-dong Zheng[1]

1 Department of Oncology, General Hospital of Shenyang Military Area Command, Shenyang 110840, P. R. China

2 Institute of Molecular Research, Shenyang University of Chemical Technology, Shenyang 110142, P. R. China

3 Department of Gynaecology and Obstetrics, First Affiliated Hospital, Jilin University, Jilin 130021, P. R. China

4 Department of Aerospace Medicine, Fourth Military Medical University, Xi'an 710032, P. R. China

5 Department of Digestive Surgery, Xijing Hospital of Digestive Diseases, The Fourth Military Medical University, Xi'an 710032, China.

6 Department of Oncology, Xijing Hospital, Fourth Military Medical University, Xi'an 710032, P. R. China

7 Rescue Center of Severe Wound and Trauma of PLA, General Hospital of Shenyang Military Area Command, Shenyang 110840, P. R. China.

*These authors have contributed equally to this work

Correspondence and requests for materials should be addressed to: Z.D.Z. (mylonzzdong@163.com), M.J.X. (manjiangxie@hotmail.com) and

M.X.H. (houmingxiao188@163.com).

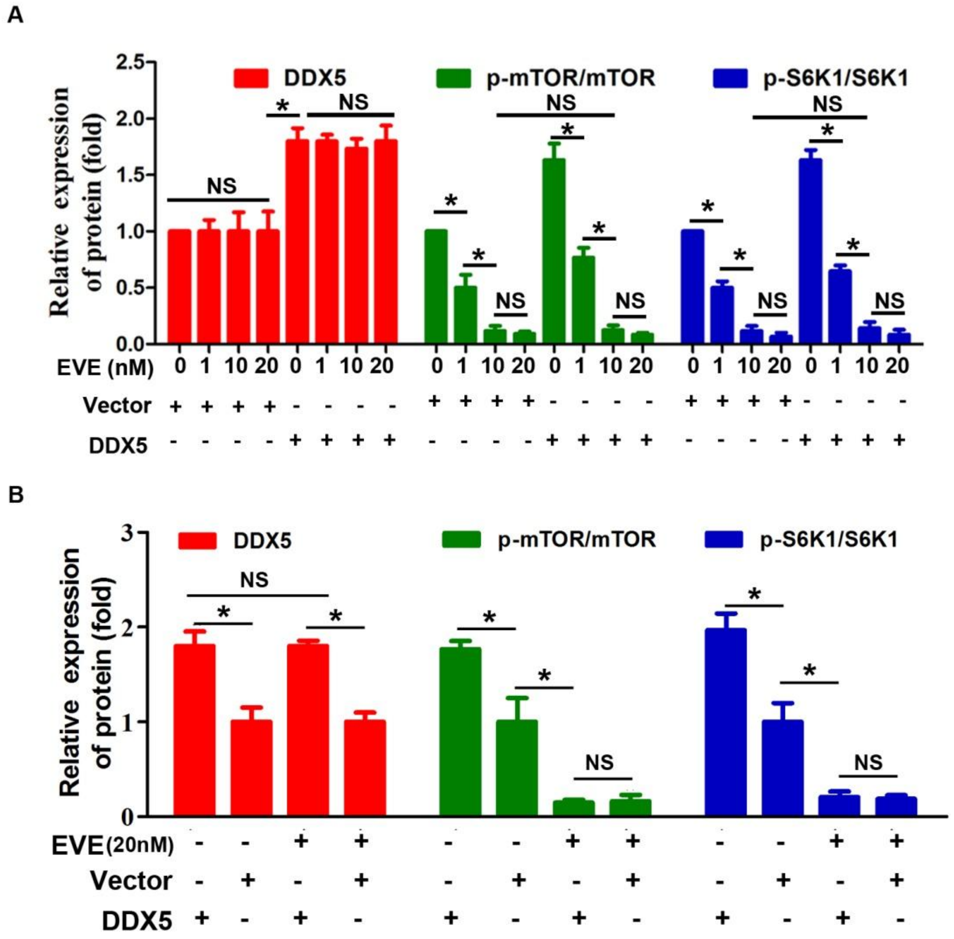

Figure S1. Relative quantification of the blots of DDX5, p-mTOR/mTOR and p-S6K1/S6K1 in (A) KATO III and (B) NCI-N87 cells treated as above indicated. (*p<0.05; NS, no significance).
